# Raising professionalism concerns as a medical student: damned if they do, damned if they don’t?

**DOI:** 10.1186/s12909-024-05144-4

**Published:** 2024-02-29

**Authors:** Erica Sullivan, Harish Thampy, Simon Gay

**Affiliations:** 1 University of Manchester, Manchester, UK; 2 University of Leicester, Leicester, UK

**Keywords:** Professionalism, Medical students, Peers, Raising concerns

## Abstract

**Background:**

Understanding professionalism is an essential component of becoming a doctor in order to ensure the trust of patients and wider society. Integrally linked to the concept of professionalism is the importance of identifying and raising concerns to ensure high quality, safe patient care. It is recognised that medical students are uniquely placed to identify and report concerns given their frequent rotations through multiple clinical placements and their peer relationships and, in so doing, develop and enact their own medical professionalism. Although there is existing literature exploring medical students’ willingness to raise concerns about observed professionalism lapses, this has largely been in the context of clinical interactions. Medical students will however undoubtedly encounter concerning behaviours or attitudes in their fellow students, an area that has not specifically been reported upon. This study therefore set out to explore medical students’ willingness to report professionalism concerns they encounter both within and away from the clinical setting, particularly focusing on peer-related concerns.

**Methods:**

10 medical students, in later clinical years of a large UK medical school, volunteered to take part in in-depth semi-structured interviews. Interviews were recorded, transcribed and then analysed thematically to generate themes and subthemes to represent central organising concepts.

**Results:**

Three broad themes were generated from the data. Hidden curricular effects including role models, hierarchical structures and the operational systems in place to raise concerns subconsciously influenced students’ decisions to raise concerns. Secondly, students offered a range of justifications to defend not taking action, including considering their own vulnerabilities and values alongside demonstrating empathy for perceived mitigating circumstances. The third theme highlighted the complex interplay of influencing factors that students considered when encountering professionalism issues in their peers including wider peer cohort effects and a desire to maintain individual peer-relationships.

**Conclusions:**

Medical students will inevitably encounter situations where the professionalism of others is brought into question. However, despite clear curricular expectations to report such concerns, these findings demonstrate that students undergo a complex decision-making process in determining the threshold for reporting a concern through navigating a range of identified influencing factors. This study highlights the important role medical schools play in helping reduce the inner conflict experienced by medical students when raising concerns and in ensuring they provide supportive processes to empower their students to raise concerns as part their own developing professionalism.

## Practice Points

Understanding professionalism is an essential component of becoming a doctor.

Identifying and raising concerns is vital to ensure high quality safe patient care.

There are many barriers to medical students raising concerns.

Medical students navigate a complex decision-making process in determining if the threshold for reporting a concern has been reached.

Medical students balance their own vulnerabilities and values, and the advantages and disadvantages of taking action when deciding whether or not to report a concern.

Medical students will additionally encounter professionalism lapses from their peers outside the clinical setting. Their willingness to subsequently raise concerns about their fellow students is considered in the light of their peer group dynamics and potential impacts on peer-relationships.

## Background

Developing professionalism in medical students is essential to ensure that future doctors are fit for clinical practice and thus engender public trust befitting of the profession. Whilst acknowledging there are many suggested definitions of what is meant by the term professionalism, in this paper we adopt the UK Royal College of Physicians perspective of professionalism being ‘a *set of values, behaviours and relationships that underpins the trust that the public has in doctors’* [1]. In the context of medical students progressing through their undergraduate training, the additional term ‘proto-professionalism’ has been coined [2] to reflect this period of time in which they acquire the knowledge, skills, attributes and experiences to attain the expected professionalism required of physicians.

An integral component of professionalism relates to identifying and raising concerns regarding observed healthcare delivery in order to promote high quality, safe patient care within transparent and supportive clinical environments [3]. In the United Kingdom, the importance of raising concerns was highlighted in the Francis Inquiry [4] which reviewed a series of failings in care that had occurred within the country’s National Health Service. The subsequent report detailed several recommendations for implementation designed to create a working culture that encouraged openness and honesty amongst all staff in raising concerns about clinical care. In particular, the report recognised that healthcare students were uniquely placed to positively contribute to this culture in view of their frequent rotations through multiple clinical placements across different health providers where they were likely to observe both good and poor examples of clinical practice.

Recognising that professionalism and raising concerns are inextricably linked issues [5], the UK’s General Medical Council (GMC) has specifically included this topic within their guidance to medical schools, encouraging directed learning within undergraduate curricula to help students understand this aspect of professional development [6]. Medical schools are therefore required to ensure that students are equipped with a clear understanding of the professional expectations others have of them; and for them, in turn, to have an expectation of the appropriate professional behaviour of others. Clinical placements are complex environments and provide real world experiential opportunities for students to further navigate their roles and developing proto-professionalism [7]. By the very nature of these complex environments, medical students will encounter situations where they identify professionalism concerns in their peers, clinical staff and the wider healthcare service team in both clinical settings and their learning environments. It is therefore crucial that medical students understand when and how to raise a concern, and feel supported in doing so.

In establishing what is already known in this regard, we recognised that the literature was rich with empirical studies exploring the issues of raising concerns across different health profession contexts (such as nursing, dentistry and medicine), training contexts (undergraduate and postgraduate) and geographical locations internationally. This wide ranging evidence-based has been analysed through several systematised reviews revealing that whilst there are many shared findings that are generalisable across different contextual settings, the phenomenon of raising concerns demonstrates context-specificity given discernible differences across disciplines and settings due to distinctive roles, responsibilities and affordances [8–11]. We therefore focussed our pre-study literature review on studies investigating raising concerns specifically among medical students in the United Kingdom.

We identified several survey-based studies exploring the factors that influenced medical students’ decisions on whether to raise a concern about observed breaches in professionalism. Johnson et al. [12] found that as students progressed into more senior years, the proportion who agreed that raising concerns was an important student responsibility fell from 91% agreement amongst year 1 students to just 79% for year 4 students. Students also reported a lackof understanding as to what constituted a concern. They experienced difficulty in judging its level of seriousness, and therefore the threshold needed for such concerns to be reported. Cited barriers to student willingness to raise concerns included the hierarchal structure of the workforce, fear of repercussions and lack of anonymity in the process. These same barriers were echoed in another UK survey of medical students with an additional influencing factor identified relating to whether students believed their concern would be acted upon [13]. Kohn et al. demonstrated from their survey that medical students weigh up the seriousness of a breach of professionalism in deciding if they should report a perceived violation with under 30% of respondents indicating they would not report minor or moderate incidents [14]. More recent findings highlight student worries that raising a concern could impact on their end-of-placement ‘sign-off’ or subsequent teaching opportunities [15]. Students indicated that they would therefore be more likely to discuss concerns with other students or junior doctors rather than formally report this to senior staff. They also expressed a need for additional training and guidance for medical students on raising concerns, improved transparency around the process and consequences of doing so, and a desire to receive feedback on actions taken following reporting.

In addition to the above survey-based exploration of raising concerns, published qualitative work in this field adds further insight into how undergraduate UK medical students navigate the challenges of reporting observed professionalism issues. A detailed analysis of 200 medical student narratives of experiencing professional dilemmas revealed a range of experienced emotional responses that arise as students navigate potential conflicts with expectations, responsibilities and their own chosen path of action [16]. Further work from the same authors, using 680 medical student narratives to focus on memorable professionalism dilemmas, revealed again a complex interplay of emotion and narrative as students struggled with disempowerment and conflicts with their own moral codes leading to feelings of distress [17].

Although there therefore exists a considerable body of evidence exploring medical students’ willingness to raise concerns, this has largely been in the context of direct clinical interactions or in light of patient safety implications. Medical students will however undoubtedly encounter concerning behaviours or attitudes that go beyond direct patient care and involving not only senior qualified staff but also fellow students. Our review of the literature has not identified that this aspect of raising concerns has been specifically previously investigated or reported. We therefore aim to address this gap through reporting results obtained from an in-depth qualitative exploration of clinical year medical students’ willingness to raise concerns about observed lapses of professionalism with a particular focus on when this arises in their peers, both within and away from the clinical environment. It explores the perceived challenges presented in raising such concerns and potential influences that determine onward action.

## Methods

### Approach

To allow for rich exploration of medical students’ experiences of this sensitive topic area, a qualitative approach using individual semi-structured interviews was selected. The authors adopted an interpretivist paradigm through the lens of social constructivism in which social phenomena are seen to occur as a part of multiple realities with meaning constructed by individuals as a result of their experiences and interactions [18]. Through in-depth interviewing, attention was paid to exploring experiences, values and attitudes pertaining to the focus of inquiry.

### Recruitment and sampling

This study was conducted at a large medical school in the UK. Ethical approval was provided by Keele University School of Medicine, at which the lead author was enrolled for Masters level studies (application reference 17 − 04).

A convenience sampling approach was adopted. Medical students in their clinical years (years 3 to 5) were invited to participate in the study through advertisements on their learning platform. Upon expressing interest, students were sent a participant information sheet detailing the study aims and requirements, before then completing a consent form. 10 students volunteered to participate as detailed in Table 1.

**Table 1:** Participant details

Gender of subject		Clinical year		
Male	Female	Year 3	Year 4	Year 5
2	8	3	5	2

### Data collection

Each student was invited to a face-to-face semi-structured interview conducted by the lead author, facilitated using a topic guide. Interviews lasted approximately 30 min each and explored students’ understandings of professionalism and experiences of raising concerns. Students were asked to share and reflect upon real examples they had personally encountered when providing responses to questions. Cognisant of the sensitivities of the topic, interviewees were assured of the confidentiality of their disclosures in order to encourage uninhibited discussions, and were made aware of independent post-interview support if required.

### Data analysis

All interviews were recorded and transcribed verbatim with all identifiers removed to preserve participant anonymity. Where applicable, identifying references to other individuals were also anonymised during transcription. Resultant transcripts were then analysed using a reflexive thematic analysis approach [19] that involved initial inductive coding to capture key or recurring aspects of the data, which were then sorted into themes and subthemes to represent central organising concepts from the data obtained.

## Results

All interviews opened with an exploration of students’ understanding of the term ‘professionalism’ to frame the later discussions on raising concerns about potential professionalism lapses. Whilst many struggled to clearly define the term, they were they were all able to identify both positive and negative professional attributes and gave examples of these from their lived experiences.

“*It’s really hard to talk about professionalism without saying the word professionalism; it’s one of those tricky concepts where everyone knows what it means…” (P1,Y4,F)*.

*“It’s kind of acting in a certain way when you’re a medical student or a doctor or in the workplace. I can’t really define it but just like being respectful and kind of following rules that we’ve been told about since starting at medical school…” (P10,Y3,F)*.

*“As a medical student I think professionalism to me is acting in like a respectful manner. So in a way that I can gain the respect of not just patients but other staff but in a way that I’m also giving that same respect back.” (P6,Y4,F)*.

Interviews thereafter focussed on discussing examples of students’ observations of poor professional practice, with a particular focus on occurrences with peers, and exploring factors that either prompted or inhibited students’ willingness to raise concerns about these. Three broad themes were generated from the data, each with subthemes (Table 2).

**Table 2:** Themes and Subthemes

Theme	Subtheme
The hidden curriculum	Role models Hierarchy Systems and processes
Justification	Personal Vulnerabilities Mitigating circumstances
Peer effects	Peer collegiality Maintaining relationships

### The hidden curriculum

This first theme highlights the unintended and unofficial norms, values and culture observed by students that influenced their willingness to raise concerns. Particular attention was paid to the effects of role models, students’ perceived position within the workplace hierarchy, and students’ beliefs about the systems and processes in place used to raise concerns.

#### Role models

Students often cited examples of the influence role models had on their own professional development. Whilst students were able to provide examples of positive professional behaviours displayed by their seniors and supervisors, they focussed more in the interviews on instances where these behaviours fell below their expectations.

*“…the patient had quite a lot of mental health problems and she was very depressed and she was crying, she was crying to us and we really didn’t know what to do so she went to see the doctor and he just basically shouted at her for not stopping smoking and said ‘you’re going to die… it was really unempathetic.”* (P1,Y4,F).

Whilst students recognised that such examples were not behaviours that they themselves would wish to emulate, they also reported subtler examples of role modelling effects in which seniors they respected and looked up to, committed perceived less serious lapses of expected standards.

*“Sometimes I understand you need to use that tactic [being directly abrupt with a patient].”* (P1,Y4,F).

Students at times struggled to reconcile these instances with their own sense of professionalism and, on occasion, would eventually view such examples as acceptable despite initial reservations, given previously held positive impressions of the senior involved.

*“…because it’s like they know better so if they’re choosing to do something then you know in their eyes it’s because they always done it or it’s the right thing to do.”* (P6,Y4,F).

#### Hierarchy

Students repeatedly voiced in the interviews how they understood their obligations to raise concerns about professionalism lapses when identified. Yet, once immersed in the clinical environment, they felt at the bottom of a hierarchal structure which inhibited their confidence to do so. Raising a concern about a senior was viewed as an adversarial process and that their role as a medical student had not yet afforded them equivalence:

*“For someone who is just a medical student to say ’actually I think this is wrong’ is scary.”* (P6,Y4,F).

*“The patient was basically crying and the consultant was on the phone the whole time, he didn’t look up at the patient once. That was very uncomfortable and I didn’t know what to do, I should be intervening to make it better for the patient but even if I don’t agree with what my supervisor is doing, I’m challenging my senior, I haven’t earned that right yet.”* (P4,Y5,M).

*“If it’s a consultant or the head surgeon, it’s the case of my voice against theirs and they’re much more experienced than me, what can I really do about that as a medical student?”* (P5,Y4,F).

Furthermore, raising concerns as a student was felt to heighten a feeling of being a burden to clinical staff, with onward impact on their learning experience.

*“…I guess they’re just gonna look down on you more than they do already or find you more annoying or irritating. We’re always told make the nurses lives easy because they know a lot and will help you… if you make their lives hard it will do the opposite.”* (P9,Y5,F).

However other participants reported their confidence and willingness to speak up to senior colleagues grew as they themselves became more senior medical students.

*“I didn’t feel that the doctor who was about to do the ABG [arterial blood gas] had explained to the patient what was about to happen and the patient was quite distressed. They asked me if I would help hold the patient. Now I would be more confident in speaking to her [the doctor about consent]… but I didn’t at the time.”* (P8,Y4,F).

#### Systems and processes

Students reported multiple examples of how their trust in the school processes to address professionalism issues and deal with concerns impacted on their decision to raise a concern. For example, when discussing fellow students who were persistently late or didn’t attend lectures, students placed trust in the school’s process for monitoring attendance to highlight these students and deal with them accordingly. Positive examples were also provided of effective processes in raising concerns that encouraged students to make disclosures:

*“At the end of our time [in the hospital] we had a big session, we got to say three things we felt were really good practice and three times we weren’t as impressed with…* (P4,Y5,M).

Others, however, reported being unfamiliar with how they would raise a concern, citing a perceived difficulty in accessing a clear and easy to use process as a barrier.

*“I assume it would mean sending an email to [named department]. I wouldn’t actually know who to contact to be honest. I think that would be another problem actually in reporting– the fact that I don’t actually know who I’d tell…”* (P7,Y3,F).

Expectations of onward outcomes following raising a concern also influenced students’ future willingness to speak up. Whilst a minority suggested they were unaware of what would happen once they had raised a concern, other students reported experiencing additional unprofessional behaviours as a result of speaking up:

*“I don’t really know what happens when I bring up concerns; I almost don’t see them as options because I don’t actually know what will happen.”* (P4,Y5,M).

*“I did mention it, [*sexist behaviour towards females on surgical placement*], to my clinical lead at the very end of the placement because I’d just been signed off and didn’t have anything to lose, he almost laughed it off and was like ‘Oh yeah, we’re trying to fix that’.”* (P5,Y4,F).

### Justification

This second theme highlights the complex internal decision-making process students undertook as they negotiated observed professionalism lapses. Students justified failing to raise concerns due to multiple interplaying factors relating to personal vulnerabilities and perceived mitigating circumstances.

#### Personal vulnerabilities

In considering whether to raise a concern, students first considered their own position in the process and their perception of the possible consequences of raising a concern. Their narratives describe both the emotive nature inherent in raising a concern and their fear of causing adverse consequences through their actions.

*“I feel that’s quite, it is quite scary. And I would be quite nervous about the response I would get back.”* (P6,Y4,F).

*“…there’s always a fear that oh what if I am in the wrong here and then if I get kicked out of medical school as a result because we’re always being told how important professionalism is.”* (P5,Y4,F).

Such fears resulted in students choosing to not speak up about concerns. Other students reflected that their own personal characteristics resulted in inaction as they chose the path of least resistance.

*“I feel guilty I didn’t say anything about it. In retrospect I probably should have done… I think the issue is that I’m quite a reserved person.”* (P5,Y4,F).

#### Mitigating circumstances

Students defended examples when they had not raised concerns despite experiencing discomfort through describing justifications that they felt mitigated negative professional behaviours they had observed. These included clinical need, and personal stressors.

*“…you can understand those things…we often see people being very stressed and snapping at one another…” (P2,Y3,F)*.

Students’ empathy towards healthcare professionals working in complex clinical environments resulted in further mitigating justification of lapses in professional standards being offered. Students described how they understood the clinical workplace as being stressful with rising workload expectations and often multiple competing demands for staff time.

“*We were supposed to have teaching and the consultants just don’t turn up, I understand they have other commitments so they’ve always had valid reasons.”* (P7,Y3,F).

*“There was this one orthopaedic consultant and in the middle of surgery there was a new nurse, she didn’t know where all the equipment was and he kept having a go at her, I felt so bad for this nurse. But you can understand why these situations arise because you expect the person to do a job properly.”* (P2,Y3,F).

Students recognised however, that there were more positive ways to manage issues that arise despite contextual and environmental demands and stressors:

[When a consultant took a junior doctor to one side to quietly discuss a mistake] *“…that was great as opposed to shouting at him in front of everybody which I would have expected him to do because of the time pressure of the theatre.”* (P7,Y3,F).

### Peer effects

In this final theme, medical students reflected upon how they navigated the challenges of raising professionalism concerns relating to their peers, both within and away from the clinical setting. Participants highlighted how they considered how their peer support networks impacted on raising concerns with a particular focus on the impact such action could have on their relationships with one another.

#### Peer collegiality

All students provided narratives describing the value they placed upon peer groups that provided mutual support as they collectively navigated through the challenges of clinical placement experiences.

*“I think there’s a sense of togetherness, we’re all in it together and other students don’t know what we’re going through, we’re quite a tight knit group which is normally a really good thing, but in that regard it probably isn’t…”* (P4,Y5,M).

However, as hinted at in the end of the above narrative, these peer support networks themselves often introduced professionalism-related situations and dilemmas outside the clinical environments that students had to further negotiate. Students recognised that the actions of their fellow students were at times at odds with the medical school’s expectations of professional behaviour, though they defended these conflicts between actions and expectations by virtue of their role as students.

*“I think there are issues about students with alcohol and drugs and think that’s kind of a big issue and I don’t think the school realise that those things go on. I don’t see it as that much of a problem doing that in their own private lives or whatever. But I know from the medical school point of view you’re doing something that’s illegal and that’s not therefore appropriate.”* (P1,Y4,F).

*“So it’s kind of balancing the thing where you say medical students have an expectation of being professional all the time and actually they are young people and need to let off a bit of steam.”* (P1,Y4,F).

However, students also provided positive examples of when peer collegiality served as a helpful trigger to speak up. The following student described doing so collectively with their peers when their views on acceptable humour conflicted with the tutor’s view.

*“We came into the classroom and there was a boy in my class who was Chinese and the tutor was like ‘I’ve got a funny video to show you, let me show you this’. It was a video of a Korean man swearing and he was laughing along saying ‘he looks like you’. We decided as a group to report him, we apologised to the boy who was involved.”* (P10,Y3,F).

#### Maintaining relationships

In discussing examples of observed professionalism lapses of peers, students reflected upon the importance, value and power they placed upon maintaining personal relationships with each another. This created ethical dilemmas that students sometimes struggled to resolve. Additionally, students recognised that their peer cohort included individuals from a wide range of backgrounds and that this influenced not only how students demonstrated professional / unprofessional behaviours but also how they dealt with situations where this was out of line with their own norms and values.

*“In anatomy people would make comments that would make me feel uncomfortable because I was raised in a culture that you have to respect the dead… everyone was laughing along.”* (P2,Y3,F).

The above student went on to describe how they were reluctant to speak up for fear of being seen to be different or not fitting in with the wider peer camaraderie.

Ultimately, despite students providing numerous examples of peer-related professionalism concerns, no participant in this study had raised these formally either to their peer or medical school staff. Indeed, there was a feeling that raising a concern about a peer could in itself be seen as unprofessional and was contrary to the strong ethos of togetherness found in their support networks. Students worried particularly about becoming unpopular, not only within their immediate circle of friends, but also in the wider group of students if they raised a concern about a fellow student.

*“… I wasn’t sure how to deal with it because there is a sense of professionalism but they are my friends and if I reported them it would have a knock on effect on our friendship.”* (P5,Y4,F).

*“I probably wouldn’t (raise a peer concern) just because I feel as though that’s up to them and I do feel like, you know, I do feel as though you would become that person that everyone knows would snitch someone up. So I probably wouldn’t.”* (P6,Y4,F).

Further defence for not raising concerns about peers was an expressed assumption that a fellow student’s personal conduct did not necessarily mean there was concern about their medical student professional role.

*“…there’s people who I’ve seen, you know just crazy people who you think I can’t believe you’re going to be a doctor in the future but then they probably are relatively normal on the ward but then their kind of home life is completely different. If he was my doctor I wouldn’t want him but I, again I’m sure he’s a good doctor.”* (P10,Y3,F).

## Discussion

Although students struggled at the start of their interviews to define professionalism, through their many examples of positive and negative professional behaviours they demonstrated a clear understanding of the professional obligations expected of medical practitioners. However, it was evident that despite a clear theoretical understanding of the importance of raising concerns there were, in practice, multiple complex factors that influenced students’ decisions to enact this expectation when professional lapses were encountered.

A variety of hidden curriculum aspects influenced students’ decisions about whether to raise a concern. The term hidden curriculum was coined to reflect that students not only learn through the planned content of the intended curriculum but through experiencing the culture, values and norms throughout their learning journey [20]. Students in this study highlighted that role models play an important part within this hidden curriculum in which they observed both positive and negative examples of professional practice. This is known to cause conscious and subconscious effects on students’ own understanding and onward display of professional expectations [21]. Consciously, students can be perceptive enough to adopt the positive elements of their role models and reject the negative [22]. However, students may also subconsciously adopt negative elements from their observed experiences as acceptable practice, despite initial concerns. This phenomenon can be considered within Lave and Wenger’s communities of practice [23] in which students move from their initial place as legitimate peripheral participants to becoming part of the central core of the workplace community through taking on expected and accepted values and attitudes. It is therefore imperative that students are provided with formal opportunities to reconcile hidden curricular experiences with expectations as they transition to centrality within their community of practice and, in doing so, deepen their own understanding of professionalism [24, 25].

Students also frequently identified that their perceived lower positioning within the hierarchy of the clinical environment served as a potential deterrent for raising concerns. Students felt uncomfortable in speaking up about those senior to them and questioned if they had sufficient rights and experience to do so. This may result in personal distress where the student attempts to balance the risk of perceived consequence with their empathy towards patients or their moral drive to do the right thing [16]. However, the emerging concept of resistance suggests that students are not always powerless and may act in the moment, (instantaneously) or later (delayed) [26]. Exercising such resistance can positively impact a culture of change and benefit students’ proto-professionalism and emerging professional identities.

Another hidden curriculum effect was students’ experiences and understanding of the systems and processes involved when raising a concern. Students expressed concern that although raising concerns was formally encouraged, they were uncertain about what actions would be taken in response. They voiced examples where they believed their concerns had not been taken seriously which then felt a contradiction to prior encouragement. This highlights the need for medical schools to ensure that their formal expectations and intended processes for managing concerns from students aligns to what then occurs in practice to itself not serve as a barrier to future reporting. Furthermore, students are more likely to be empowered to raise a concern when they are confident that their action will make a difference, a phenomenon that Johnson et al. [12] refer to as ‘conviction’. It is therefore imperative that feedback is provided to students who raise concerns about what has occurred and to highlight the role they have played in improving standards.

Students also reported a complex interaction of factors that served as justification to defend examples when they had not acted in raising a concern. Students worried about the personal implications of doing so and often first considered the potential for negative repercussions when deciding how to act. Students also feared the personal impact that raising a concern would have on their own learning opportunities and subsequent progression. These anxieties around raising concerns have been consistently demonstrated in other work exploring not just medical students, but nursing students and junior doctors [11, 27]. The Francis report [4] recognises this student vulnerability and recommends that proactive steps must be taken to ensure they are supported when raising concerns.

Students’ justifications for not raising concerns extended beyond personal considerations into expressing understanding and empathy that there were at times mitigating circumstances that meant that what they understood about professional expectations in the classroom, may not always be possible to enact in the stressful, busy and uncertain environment of the clinical workplace.

This study adds specific insight into the under-researched challenges of medical students raising concerns relating to their peers. Students valued their peer networks and utilised these as a way to support each other through both enjoyable and difficult experiences. However, students were generally unwilling to escalate concerns about a fellow student beyond their immediate peer group. This contrasts with findings from a USA-based study in which the majority of surveyed medical indicated they would report peers who demonstrated concerning professional behaviour [28]. In our study, students’ reluctance to speak up about peers were driven by fears of jeopardising relationships and aims to preserve self-image and reputation, similar to findings observed in nursing students [29]. This was in part attributable to a desire to ‘fit in’ with their peers, even when students appreciated that there was conflict between their own values and those that they observed. Similar findings have been demonstrated in other work exploring how medical students negotiate professional dilemmas and how this informs their emerging professional identities [30]. In attempting to ‘fit in’, students experienced conflicts between their own sense of professionalism with what they observed, with a variable threshold for triggering a decision to raise a concern dependent on the incident and context. As such, their decision making was not a passive process but instead was inherently emotional, invoking feelings of self-doubt, worry, fear and ultimately guilt when inner feelings were not translated into actions. Suggested interventions to help address such experienced moral distress use formal opportunities for students to freely discuss professional dilemmas they have encountered [16]. Through facilitated reflection, students can be helped to make sense of situations and develop their professional identity in a way that inculcates professionalism as its core.

The nature and context of a concern about a peer also influenced the threshold for when to escalate this further. For example, students in this study suggested they were more forgiving of lapses in a peer’s personal conduct over those related to the medical student professional role. Yet in other work, academic misconduct of a peer was rarely reported by students despite the majority of them recognising the need to do so [31].

### Reflections

The authors recognise that this work was conducted at a single medical school and that students were self-selecting volunteers and so may have held views unrepresentative of the wider student population. However, through recruiting ten students across clinical year groups, we successfully generated a rich data set to create broad themes that has applicability across differing UK medical school contexts. The lead researcher, who conducted the interviews, is currently a medical school academic and so it is acknowledged their position of authority may have impacted upon the students’ responses. However, all attempts were made to ensure that students felt uninhibited in making disclosures, with students reassured throughout that anonymity would be maintained and reiteration that they would not face any negative repercussions through involvement in this work.

Whilst this study has provided detailed understanding of the myriad of enablers and barriers relating to raising concerns, there remain unanswered questions. This work did not specifically explore the influence that student demographics, such as gender and ethnicity, and culture, had on them raising concerns and so additional work is needed to understand this further. Secondly, the focus of this work was on understanding the complex interplay of factors that influenced decision making in raising concerns over exploring potential solutions to negate barriers. Further intervention-based studies are therefore needed to identify effective and practical options to help encourage students in raising concerns specifically in relation to peer-related concerns.

## Conclusions

Raising concerns is an inextricable component of professional clinical practice that has attracted particular attention given well-publicised examples of failures to promote safe patient care. Subsequent review recommendations have recognised that healthcare students are well-placed to identify and report lapses in expected professional behaviours of others. Yet despite these expectations, this study has identified a range of factors that influence medical students’ willingness to raise concerns.

This study adds to current knowledge in this area through moving beyond the existing focus of prior work on concerns in the context of patient safety by considering raising concerns about professional lapses encountered away from the clinical setting and particularly, those involving peers. This study’s findings suggest that as medical students progress through their clinical years, they navigate a complex decision-making process in determining if the threshold for reporting a concern has been reached. This involves them considering their own vulnerabilities and values alongside balancing the advantages and disadvantages of taking action through perceived justification of each approach, all of which is influenced by hidden curriculum effects such as role models and observed operationalisation of the raising concerns process itself.

Ultimately, these findings mean that medical schools would be wise to review their current processes and teaching in this area to ensure that their students are truly empowered to raise concerns as part of enacting their own sense of professionalism.

## Data Availability

De-identified data can be made available upon request. To request the de-identified data please contact the corresponding author Mrs Erica Sullivan erica.sullivan@manchester.ac.uk.

## References

[1] RCP (2005). Doctors in society: medical professionalism in a changing world. Report of a Working Party of the Royal College of Physicians of London.

[2] Hilton SR, Slotnick HB (2005). Proto-professionalism: how professionalisation occurs across the continuum of medical education. Med Educ.

[3] GMC. Good Medical Practice: General Medical Council.; 2013 [Available from: https://www.gmc-uk.org/ethical-guidance/ethical-guidance-for-doctors/good-medical-practice.

[4] Francis R. Report of the Mid Staffordshire NHS Foundation Trust public inquiry: executive summary. The Stationery Office; 2013.

[5] Hendelman W, Byszewski A (2014). Formation of medical student professional identity: categorizing lapses of professionalism, and the learning environment. BMC Med Educ.

[6] GMC. Medical professionalism matters. General Medical Council; 2016.

[7] Ramani S, Leinster S (2008). AMEE Guide 34: teaching in the clinical environment. Med Teach.

[8] Okuyama A, Wagner C, Bijnen B (2014). Speaking up for patient safety by hospital-based health care professionals: a literature review. BMC Health Serv Res.

[9] Violato E (2022). A state-of-the-art review of speaking up in healthcare. Adv Health Sci Educ Theory Pract.

[10] Jones A, Blake J, Adams M, Kelly D, Mannion R, Maben J (2021). Interventions promoting employee speaking-up within healthcare workplaces: a systematic narrative review of the international literature. Health Policy.

[11] Milligan F, Wareing M, Preston-Shoot M, Pappas Y, Randhawa G, Bhandol J (2017). Supporting nursing, midwifery and allied health professional students to raise concerns with the quality of care: a review of the research literature. Nurse Educ Today.

[12] Johnson L, Malik N, Gafson I, Gostelow N, Kavanagh J, Griffin A (2018). Improving patient safety by enhancing raising concerns at medical school. BMC Med Educ.

[13] Kakkar A, Lynch D (2019). Medical students raising concerns about staff members. Future Healthc J.

[14] Kohn JR, Armstrong JM, Taylor RA, Whitney DL, Gill AC (2017). Student-derived solutions to address barriers hindering reports of unprofessional behaviour. Med Educ.

[15] Druce MR, Hickey A, Warrens AN, Westwood OMR (2021). Medical students raising concerns. J Patient Saf.

[16] Monrouxe LV, Rees CE (2012). It’s just a clash of cultures: emotional talk within medical students’ narratives of professionalism dilemmas. Adv Health Sci Educ.

[17] Rees CE, Monrouxe LV, McDonald LA (2013). Narrative, emotion and action: analysing ‘most memorable’ professionalism dilemmas. Med Educ.

[18] Kim B. Social constructivism. Emerging perspectives on learning, teaching, and technology. 2001;1(1):16.

[19] Braun V, Clarke V (2019). Reflecting on reflexive thematic analysis. Qualitative Res Sport Exerc Health.

[20] Hafferty FW (1998). Beyond curriculum reform: confronting medicine’s hidden curriculum. Acad Med.

[21] Passi V, Johnson S, Peile E, Wright S, Hafferty F, Johnson N (2013). Doctor role modelling in medical education: BEME Guide 27. Med Teach.

[22] HCPC. Professionalism in healthcare professions. Health and Care Professions Council; 2014.

[23] Lave J, Wenger E. Situated learning: legitimate peripheral participation. Cambridge University Press; 1991.

[24] Monrouxe LV, Rees CE, Hu W (2011). Differences in medical students’ explicit discourses of professionalism: acting, representing, becoming. Med Educ.

[25] Farrington R, Collins L, Fisher P, Danquah A, Sergeant J (2019). Clinical debrief: learning and well-being together. Clin Teach.

[26] Shaw MK, Rees CE, Andersen NB, Black LF, Monrouxe LV (2018). Professionalism lapses and hierarchies: a qualitative analysis of medical students’ narrated acts of resistance. Soc Sci Med.

[27] Hooper P, Kocman D, Carr S, Tarrant C (2015). Junior doctors’ views on reporting concerns about patient safety: a qualitative study. Postgrad Med J.

[28] Hodges LE, Tak HJ, Curlin FA, Yoon JD (2016). Whistle-blowing in Medical School: a national survey on peer accountability and Professional Misconduct in Medical Students. Acad Psychiatry: J Am Association Dir Psychiatric Resid Train Association Acad Psychiatry.

[29] Ion R, Smith K, Moir J, Nimmo S (2016). Accounting for actions and omissions: a discourse analysis of student nurse accounts of responding to instances of poor care. J Adv Nurs.

[30] Monrouxe LV (2009). Negotiating professional identities: dominant and contesting narratives in medical students’ longitudinal audio diaries. Curr Narratives.

[31] Rennie SC, Crosby JR (2002). Students’ perceptions of whistle blowing: implications for self-regulation. A questionnaire and focus group survey. Med Educ.

